# Comparative genomic analyses identify common molecular pathways modulated upon exposure to low doses of arsenic and cadmium

**DOI:** 10.1186/1471-2164-12-173

**Published:** 2011-04-01

**Authors:** Margaret Ann Benton, Julia E Rager, Lisa Smeester, Rebecca C Fry

**Affiliations:** 1 Department of Environmental Sciences and Engineering, Gillings School of Global Public Health, University of North Carolina, Chapel Hill, NC, USA

## Abstract

**Background:**

Exposure to the toxic metals arsenic and cadmium is associated with detrimental health effects including cancers of various organs. While arsenic and cadmium are well known to cause adverse health effects at high doses, the molecular impact resulting from exposure to environmentally relevant doses of these metals remains largely unexplored.

**Results:**

In this study, we examined the effects of *in vitro *exposure to either arsenic or cadmium in human TK6 lymphoblastoid cells using genomics and systems level pathway mapping approaches. A total of 167 genes with differential expression were identified following exposure to either metal with surprisingly no overlap between the two. Real-time PCR was used to confirm target gene expression changes. The gene sets were overlaid onto protein-protein interaction maps to identify metal-induced transcriptional networks. Interestingly, both metal-induced networks were significantly enriched for proteins involved in common biological processes such as tumorigenesis, inflammation, and cell signaling. These findings were further supported by gene set enrichment analysis.

**Conclusions:**

This study is the first to compare the transcriptional responses induced by low dose exposure to cadmium and arsenic in human lymphoblastoid cells. These results highlight that even at low levels of exposure both metals can dramatically influence the expression of important cellular pathways.

## Background

Arsenic and cadmium are ranked among the top ten priority hazardous substances by the Agency for Toxic Substances and Disease Registry (ATSDR) [1]. Exposure to arsenic and cadmium can lead to adverse health outcomes such as lung and kidney cancers as well as cardiovascular disease and diabetes [2,3]. Further, exposure to these two toxic and well-classified chemicals is of particular interest because of their extensive global impact [4-6]. For example, it is estimated that more than 40 million people worldwide drink water containing arsenic at concentrations that exceed the World Health Organization (WHO) and Environmental Protection Agency (EPA) drinking water guideline of 10 ppb [7]. Also, humans are exposed to low levels of cadmium through food consumption, typically ranging between 8 and 25 ug per day [3]. Smoking populations experience higher levels of cadmium exposure, as one cigarette may contain 1-2 ug cadmium [3].

Both arsenic and cadmium are classified as Group 1 carcinogens by the International Agency for Research on Cancer (IARC) [4,5]. Arsenic exposure has been linked to several types of cancer, including skin, lung, liver, and bladder [5,8]. Proposed mechanisms of arsenic-induced disease include oxidative stress, DNA repair inhibition, and epigenetic events [8]. Likewise, cadmium exposure has been associated with various cancers, such as prostate, kidney, pancreas, and lung [3]. The probable mechanisms of cadmium carcinogenesis are similar to those of arsenic and include aberrant gene expression, inhibition of DNA damage repair and apoptosis, and oxidative stress [9]. Studies suggest that even at low levels, chronic exposure to arsenic and cadmium is associated with increased risk for disease including cancer [10,11]. However, the exact mechanisms that associate low level exposures of arsenic and cadmium with many of these negative health outcomes remain largely unknown.

In this study, we set out to contrast the cellular responses of human TK6 lymphoblastoid cells upon exposure to low, environmentally relevant doses of arsenic and cadmium. The rationale for this research was based on studies suggesting that differential gene expression occurs in human cells following exposure to low levels of either arsenic [12-15] or cadmium [16]. Here we characterized the effects of low dose exposure to either arsenic or cadmium by examining changes in the expression of genes and their associated biological pathways and functions. We found that while the modified gene sets were distinct for each of the two metals, similar biological pathways were modulated between the two. Future research will extend these findings to identify modulated protein activity associated with such low dose exposure.

## Results

### Metal-induced gene sets identified

In this study, human TK6 lymphoblastoid cells were exposed to either arsenic (sodium arsenite) or cadmium (cadmium chloride) at low, equimolar doses (0.1 μM) for 24 hours (see Methods). These metal concentrations were minimally cytotoxic with 99% cell survival for both arsenic and cadmium (Table 1). RNA was extracted from metal-exposed or mock-treated control cells and hybridized to Affymetrix GeneChip^® ^Human Gene 1.0 ST arrays (see Methods). Differentially expressed genes were identified using an ANOVA model (see Methods). A total of 167 genes (209 probesets) were significantly differentially expressed in the metal-exposed cells; 62 of these genes were dysregulated in the arsenic-exposed cells and 105 in the cadmium exposed cells (Table 1 see Additional File 1: Arsenic-modulated genes, Additional File 2: Cadmium-modulated genes). It should be noted that the two metal-induced gene sets were distinct with no overlap between them.

**Table 1:** Summary of biological responses of TK6 cells exposed to arsenic or cadmium

	Arsenic (0.1 μM)	Cadmium (0.1 μM)
Percent Survival	99%	99%
Genes Differentially Expressed (probesets)	62 (74)	105 (135)

Gene Set Enrichment (FWER)	Cancer Module 88 *(Liver, heart, lung) (0.33)*	Cancer Module 88 *(Liver, heart, lung) (0.49)*
	
	Cancer Module 55 *(Liver, lung, breast) (0.33)*	Cancer Module 55 *(Liver, lung, breast) (0.49)*

		Cancer Module 23 *(Liver, lung, prostate) (0.49)*

		Cancer Module 6 *(Lung, prostate, liver) (0.34)*

		

Transcription Factor Binding Site Enrichment (p-value)	RBP-Jkappa (0.001)	ZF5 (0.002)
	
	Oct-1 (0.002)	Sp1 (0.011)

	E2F (0.015)	SREBP-1 (0.016)

### Metal-induced molecular networks enriched for numerous biological processes

To identify molecular networks associated with low dose arsenic or cadmium exposure, we analyzed the metal-modulated gene sets for known interactions among their encoded proteins (see Methods). For the arsenic gene set, 41 of the 62 differentially expressed genes were eligible for network generation (see Additional File 3: Gene products in networks). For the cadmium gene set, a total of 71 of the 105 differentially expressed genes were eligible for network generation (see Additional File 3: Gene products in networks).

**Figure 1:**
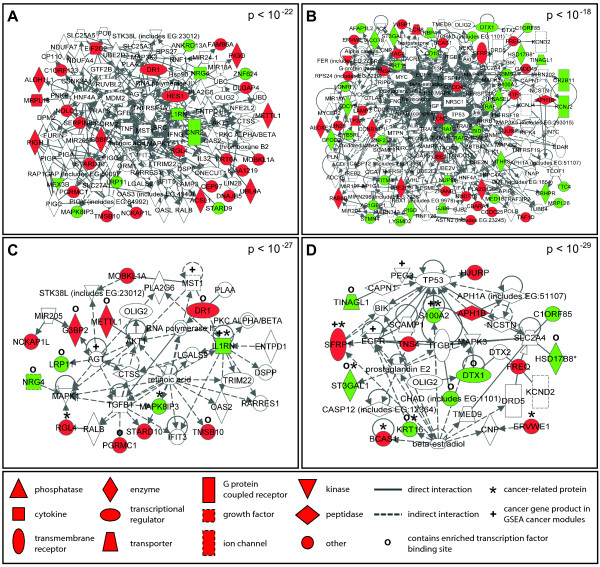
**Molecular interactomes and sub-networks modulated in TK6 cells exposed to arsenic or cadmium**. Two large interactomes were identified in TK6 cells exposed to (A) arsenic or (B) cadmium. The two most significant cancer-enriched sub-networks within the interactomes were identified for (C) arsenic and (D) cadmium. Networks are displayed with symbols representing encoded proteins corresponding to their RNA transcripts that were either directly up-regulated (red symbols), down-regulated (green symbols), or associated with the modified transcripts (while symbols). P-values representing network significance are shown.

Through network analysis we identified that exposure to either arsenic or cadmium exposure results in the modulation of large interacting protein networks (e.g. interactomes). The arsenic-induced interactome was comprised of a total of 103 proteins, 35 of which were encoded by transcripts that were differentially expressed following exposure (p < 10^-22^, Figure 1A, see Additional File 3: Gene products in networks). Within this arsenic-modulated interactome, 26 genes showed increased expression and 9 genes showed decreased expression as a result of arsenic exposure (Figure 1A, see Additional File 3: Gene products in networks).

The cadmium-modulated interactome contained a total of 167 proteins with significant interactions (p < 10^-18^); 64 of these proteins were encoded by transcripts that were differentially expressed following exposure (Figure 1B, see Additional File 3: Gene products in networks). Within this large cadmium-modulated interactome, the expression of 29 genes was up-regulated and the expression of 35 genes was down-regulated (Figure 1B, see Additional File 3: Gene products in networks).

**Figure 2:**
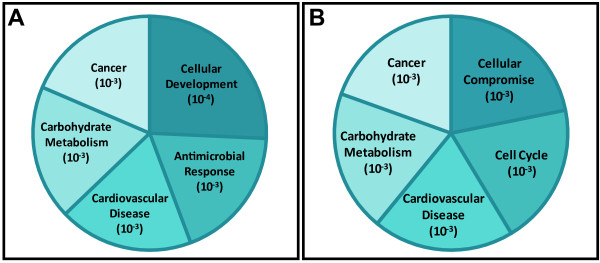
**Biological functions enriched in molecular networks altered in TK6 cells exposed to arsenic or cadmium**. The five most significantly enriched biological functions within the (A) arsenic or (B) cadmium-induced molecular networks are illustrated.

Within the large cadmium and arsenic-induced interactomes were smaller, more focused sub-networks. The arsenic interactome was comprised of three sub-networks with p values ranging from 10^-27 ^to 10^-22 ^(Figure 1C, see Additional File 4: Metal modulated sub-networks). These arsenic-modulated sub-networks were enriched for biological processes such as cellular development (p < 10^-4^), antimicrobial response (p < 10^-3^), carbohydrate metabolism (p < 10^-3^), cardiovascular disease (p < 10^-3^), and cancer (p < 10^-3^) (Figure 2A). Contained within the large interactome were genes with known associations with cancer development and progression. These included interleukin 1 receptor antagonist (*IL1RN*), mitogen-activated protein kinase 8 interacting protein 3 (*MAPK8IP3*), ral guanine nucleotide dissociation stimulator-like 4 (*RGL4*), cannabinoid receptor 2 (*CNR2*) and lin-28 (*LIN28*).

As was seen in response to arsenic, we identified significant sub-networks in the cadmium-modulated interactome with p values ranging from 10^-31 ^to 10^-18 ^(Figure 1D, see Additional File 4: Metal modulated sub-networks). The five sub-networks are enriched for processes involving cellular compromise (p < 10^-3^), carbohydrate metabolism (p < 10^-3^), cardiovascular disease (p < 10^-3^), cell cycle functions (p < 10^-3^), and cancer (p < 10^-3^) (Figure 2B). In the cadmium-modulated interactome and within the significant sub-networks, many proteins with known roles in tumorigenesis were identified. Such proteins include breast carcinoma amplified sequence 1 (BCAS1), endogenous retroviral family W, env(C7), member 1 (ERVWE1), keratin 16 (KRT16), S100 calcium binding protein A2 (S100A2), secreted frizzled-related protein 1 (SFRP1), and ST3 beta-galactoside alpha-2,3-sialyltransferase 1 (ST3GAL1), and actin filament associated protein 1-like 2 (AFAP1L2). These analyses also show the enrichment of transcripts that encode proteins involved in pathways regulated through nuclear factor kappa-light-chain-enhancer of activated B cells (NF-κB) and p38 mitogen-activated protein kinase (p38 MAPK) (see Additional File 4: Metal modulated sub-networks, Figure E).

While the arsenic and cadmium-induced gene sets shared no genes in common, we set out to establish whether the metal-modulated genes map to common pathways. For this, we combined the arsenic and cadmium gene lists and analyzed the resulting metal-modulated molecular networks. Interestingly, the networks showed overlap with both arsenic and cadmium-associated gene products mapping to common pathways including the p38 mitogen-activated protein kinase (p38 MAPK) and hepatocyte nuclear factor 4 (HNF-4) pathways (see Additional File 4: Metal modulated sub-networks).

### Cancer modules enriched in the metal-modulated gene sets

As an alternative approach to identify biological pathways that are modulated upon exposure to arsenic or cadmium, we performed gene set enrichment analysis (see Methods). We compared the metal-modulated gene sets to genes known to be dysregulated under certain physiological and environmental conditions (e.g. cancer modules) [17]. These results showed significant enrichment for two cancer modules common to both arsenic and cadmium: cancer modules 88 and 55 which include gene sets associated with liver, lung, heart, breast, and prostate cancer (Table 1). In addition, the cadmium-modulated gene set was uniquely enriched for cancer modules 6 and 23 (Table 1).

### Transcription factors identified that may mediate metal-induced transcriptional responses

To identify regulatory mechanisms that potentially underlie the metal-modulated transcript levels, we investigated whether binding sites for specific transcription factors were computationally enriched in the promoter regions of the metal-modulated gene sets (see Methods). In the arsenic-induced gene set, computational analysis of the promoter regions of the differentially expressed genes identified a significant enrichment for the following two transcription factors: EF2 transcription factor (E2F) (p < 0.015), octamer-1 transcription factor (Oct-1) (p < 0.002) and RBP-Jkappa transcription factor (p < 0.001) (Table 1). In the cadmium-modulated gene set, the enriched transcription factors included sp1 transcription factor (Sp1) (p < 0.011), sterol regulatory element binding transcription factor 1 (SREBP-1) (p < 0.016), and zinc finger protein 161 homolog (ZF5) (p < 0.002) (Table 1). A comparison of the predicted gene targets of these transcription factors shows the enrichment for their putative activity in the cancer-associated proteins in the cadmium sub-network. Specifically, ST3 beta-galactoside alpha-2,3-sialyltransferase 1 (ST3GAL1) is predicted to be regulated by ZF5 and Sp1. Likewise, keratin 16 (KRT16) is predicted to be regulated by Sp1 (see Additional File 5 Figure 1).

### Microarray gene expression results validated using qRT-PCR

Within each of the metal-modulated interactomes, target genes were selected and analyzed for their expression levels using quantitative real-time PCR (qRT-PCR). Selected from the arsenic-modulated networks, qRT-PCR analysis showed that StAR-related lipid transfer (START) domain containing 9 (*STARD9*) was down-regulated by arsenic exposure (-1.31 fold change (FC)), and methyltransferase like 1 (*METTL1*) was up-regulated (+1.46 FC). Likewise, qRT-PCR analysis showed that cannabinoid receptor 2 (*CNR2*) was down-regulated (-1.26 FC). A comparison of the fold changes obtained with qRT-PCR with those obtained by microarray shows a high correlation (0.87) (see Additional File 6).

Selected from the cadmium interactome, qRT-PCR showed that actin filament associated protein 1-like 2 (*AFAP1L2*) was down-regulated (-0.76 FC,), and lin-28 homolog (*LIN28*) was up-regulated (+4.0 FC,) following low dose cadmium exposure. qRT-PCR analysis showed that S100 calcium binding protein A2 (*S100A2*) was down-regulated (-1.27 FC). In order to confirm that metallothionein genes were not differentially expressed upon exposure to low-dose arsenic or cadmium, metallothionein 1A (*MT1A*) was assessed and found to be insignificant (p value ≥ 0.3). A comparison of the fold changes obtained with qRT-PCR with those obtained by microarray shows a high correlation (0.92) (see Additional File 6).

## Discussion

In this study we set out to compare the transcriptional responses and associated biological pathways modulated in TK6 cells exposed to low levels of arsenic or cadmium. Our aim was to contrast the genomic responses of TK6 cells exposed to either of these metals at environmentally relevant doses. Our rationale for the selection of TK6 lymphoblastoid cells is that they are well characterized at the molecular level and have been used to analyze cellular responses upon exposure to a host of environmental agents, including arsenic [18-22]. Using the most recently developed Affymetrix ST arrays that more fully cover open reading frames across the genome, we established the differential gene expression between untreated and metal-exposed TK6 cells. The transcriptional effect of exposure was greater for the cells exposed to cadmium than for arsenic with 105 and 62 genes, respectively. It was interesting to find that there were no genes in common between the two gene sets identified. The microarray gene expression results were validated using real time PCR and a high correlation (0.87-0.92) was found.

Using network analysis, we evaluated the effects of exposure to the two metals by overlaying the differentially expressed genes onto known molecular interaction maps and identified two large interactomes of inter-connected proteins. The most significantly enriched cellular functions within these networks included processes related to cancer, carbohydrate metabolism, and cardiovascular disease. Not surprisingly as their gene sets were distinct, there were also biological functions that were unique to TK6 cells exposed to either arsenic or cadmium. For example, TK6 cells exposed to arsenic modulated biological pathways associated with cellular development and antimicrobial response, whereas TK6 cells exposed to cadmium modulated cellular compromise and cell cycle functions. It is important to note that while the arsenic and cadmium-induced gene sets were distinct with no overlap in genes, the metal-modulated genes do map to common pathways. Specifically, the arsenic and cadmium-associated gene products both map to the p38 MAPK and HNF-4 pathways.

### Arsenic exposure results in the modulation of transcripts associated with cancer, cell proliferation, cell death, and inflammation

Within the arsenic-modulated interactome, there were gene products with known associations to cancer, cell proliferation, cell death, and inflammation. These include the protein products CNR2, IL1RN, LIN28, MAPK8IP3, and RGL4. *CNR2*, found to be down-regulated through both qRT-PCR and microarray analysis, has several anticancer functions. For example, it inhibits cell proliferation, reduces angiogenesis, reduces cell migration and metastasis, and attenuates inflammatory processes [23]. *IL1RN*, here down-regulated by arsenic exposure, is known to inhibit the activity of IL-1, a major human cytokine implicated in inflammatory responses that contribute to tumor development [24]. Evidence also suggests that IL1RN activity may play an important role in the development of cervical squamous cell carcinoma [25]. Further, in a chronically arsenic exposed population, Argos *et al*. found that *IL1RN *was down-regulated among individuals with skin lesions compared to those without lesions [26]. Our microarray results also highlight the up-regulation of *LIN28 *further validated through qRT-PCR. LIN28 has known associations with cancer, as decreased transcription decreases cancer metastasis [27].

### Cadmium exposure results in the alteration of the expression of genes involved in tumorigenesis, cell cycle, apoptosis, and oxidative stress

The cadmium-modulated interactome contains gene products with known involvement in tumorogenesis, cell cycle, apoptosis, and oxidative stress. For example, *AFAP1L2 *showed decreased expression levels as assessed through both qRT-PCR and microarray analysis. *AFAP1L2 *has been identified as one of six potential gene markers of tumors exhibiting local aggressiveness [28]. Also, *S100A2 *showed decreased transcript levels in TK6 cells exposed to cadmium assessed through both qRT-PCR and microarray analysis. S100A2 is a known tumor suppressor that controls tumor progression and growth [29,30]. *S100A2 *differential expression has been observed in cancers such as laryngeal squamous cell carcinoma [31] and non-small cell lung cancer [32]. Another cancer-associated gene, *SFRP1*, is up-regulated in TK6 cells exposed to cadmium. SFRP1 is an antagonist of Wnt signaling and is involved in apoptosis, cell differentiation, and signal transduction [33,34]. A previous association between the Wnt signaling pathway and cellular responses to cadmium exposure has been found [35]. *ST3GAL1*, up-regulated by cadmium exposure, is differentially expressed in several types of cancer including bladder [36], ovarian [37], breast [38], and colorectal [39,40]. We note that these genes are involved in cellular mechanisms that control cell growth and proliferation and here they are altered in TK6 cells exposed to low doses of cadmium.

Cadmium exposure has been associated with the activation of oxidative stress pathways [9]. Our findings support that oxidative stress responsive genes and potential regulatory pathways are altered in TK6 cells exposed to low doses of cadmium. Here we find the *S100A2 *gene is modulated by exposure to cadmium. *S100A2 *is associated with oxidative stress in keratinocytes [41], and is down-regulated by ultraviolet B exposure, possibly in response to free radical activity [42]. In addition, our pathway analysis shows the enrichment for NF-κB and p38 MAP kinase-associated proteins altered by cadmium exposure. Both of these molecules have been shown to regulate genes associated with oxidative stress [43].

### Gene Set Enrichment Analysis shows that common cancer gene sets are modulated by environmentally relevant levels of arsenic and cadmium

We performed Gene Set Enrichment Analysis (GSEA) to determine whether transcripts with common biological functions were differentially expressed in the metal-treated cells. Four cancer-related gene expression signatures (modules) altered by arsenic or cadmium were identified. Two of the signatures were common for both the arsenic and cadmium-induced gene sets. Specifically, cancer modules 88 and 55 were enriched in both gene sets. Cancer module 55 resembles a gene expression profile present in various tumors including liver, lung, B lymphoma, and breast cancer. Cancer module 88 resembles a gene expression profile observed in various tumors including those of the liver, lung and breast. The enrichment of cancer module 6 in the cadmium-induced gene set represents a cancer signature identified in liver, lung, prostate, and breast cancers and also leukemia. The cadmium-modulated genes were similar to those that control liver functions, such as metabolism (module 23), that are associated with liver, lung, prostate, and breast cancer and leukemia and B lymphoma [44]. It is interesting to note again that while no genes were commonly dysregulated between the two metal exposures, here the gene set enrichment analysis identified common cancer-associated modules within both. Thus, this analysis suggests that even low dose environmentally relevant exposures to arsenic or cadmium can induce similar gene expression signatures, reflective of those seen in several types of cancer.

### Promoter sites of metal-modulated genes are enriched for transcription factors involved in cell cycle regulation

To identify transcription factors that may mediate the transcriptional responses in TK6 cells exposed to arsenic or cadmium, we employed computational prediction analysis of transcription factor binding sites for both metal-induced gene sets. Computational analysis was used to identify transcription factors predicted as regulators of the observed molecular response. Analysis of the promoter regions of the arsenic-modulated genes showed enrichment for E2F, Oct-1, and RBP-JKappa. E2F regulates the expression of genes that generate proteins required for cell cycle progression, cell proliferation, apoptosis, and cell differentiation [45,46]. Oct-1 is induced by DNA damaging therapeutic agents through a posttranscriptional mechanism that does not require the tumor suppressor p53, suggesting that Oct-1 may play an important role in p53-independent gene activation to regulate cellular responses to DNA damage [47]. It has been shown that RBP-JKappa is a repressor of NF-κβ2 transcription, which regulates the expression of genes involved in cell cycle regulation and inflammation [48].

Transcription factor binding site enrichment analysis of the promoter regions of the cadmium-modulated genes showed significant enrichment for transcription factors Sp1, SREBP-1, and ZP5. Sp1, an important mediator of the cell cycle [49], may activate IL-10 in response to inflammatory signals [50] and play a role in the cellular responses to DNA damage and tumor metastasis [51,52]. Sp1 also has known involvement in oxidative stress response pathways [43]. Interestingly, Sp1 is a known regulator of two cancer-associated genes in the cadmium-associated networks. Specifically, ST3 beta-galactoside alpha-2,3-sialyltransferase 1 (*ST3GAL1*) and keratin 16 (*KRT16*) are regulated by Sp1. The Sterol regulatory element-binding protein-1 (SREBP-1) regulates the expression of lipogenic genes, such as Fatty acid synthase (*FAS*) [53]. SREBP-1 activity has been seen in both colorectal neoplasia and breast cancer [54]. Although no common transcription factors were identified between the arsenic and cadmium-modulated gene sets, the induced transcriptional changes of the target genes are associated with similar biological processes.

### Comparison of metal-induced genomic response of TK6 cells to existing genomic studies

In an effort to gain further understanding of the relevance of these findings, we compared the genes and pathways identified here to existing genomic studies. Specifically, we compared our findings to the gene expression changes that were observed in a human population in Thailand exposed to arsenic at varying levels [55]. In that study, a total of 447 genes with altered expression were associated with arsenic exposure. Two of the genes, namely *IL1RN *and *G3BP2*, were also found to be differentially expressed here in TK6 cells exposed to low-dose arsenic. As discussed previously, IL1RN is a human cytokine involved in inflammation and tumorigenesis [24]. Also, *G3BP2 *is often over-expressed in human cancers, and has been suggested to act as a negative regulator of the p53 tumor suppressor pathway [56]. In the previous study, enrichment for NF-κB binding sites in the arsenic-modulated genes was identified. Interestingly, here we find enrichment for NF-κB-associated genes altered in response to low-dose cadmium exposure, but not to arsenic.

We further compared the results obtained here with the metal-induced transcriptional response in yeast exposed to various transition metals [57]. In the study by Jin *et al*. the investigators exposed yeast to arsenic and cadmium and compared the transcriptional responses at various concentrations [57]. Two genes modified here in response to arsenic exposure, namely *C1ORF123 *and *DNAJB5*, were also found to have altered expression in yeast exposed to arsenic. There were no genes with common transcriptional response between the TK6 cells and yeast exposed to cadmium. These genomic comparisons highlight some commonalities in the transcriptional changes induced by arsenic and cadmium exposure, but the larger lack of overlap suggests that different transcriptional responses are likely influenced by cell type, organism, and dose.

## Conclusions

In this study, we identified genes with expression levels that were significantly changed in TK6 cells exposed to low doses of either arsenic or cadmium. Our selection of these two metals was based on their environmental prevalence and our interest in identifying biological processes that may be modulated by low level exposure. Using a systems level approach to examine genes and their associated networks, we show that even low-level exposure to these metals impacts gene expression. In addition, both metals were found to modulate genes that encode proteins with similar biological functions in the cell. These results highlight that even at low doses, exposure to either of these metals has an impact on the expression of molecular networks associated with important biological functions. These results suggest that more research is needed to examine the biological effects of low dose exposure to toxic metals as such pathways are potentially modulated not only *in vitro*, but modulated *in vivo *as well. Future research will include the examination of protein activity associated with the metal-modulated genes and predicted transcription factors. In addition, these findings suggest that studies examining samples from humans with known low-level exposures to these metals would be warranted and results compared to our *in vitro *studies.

## Methods

### Cell Type

The human TK6 lymphoblastoid cell line was obtained from ATCC (Manassas, VA) and cultured according to ATCC's guidelines (RPMI 1640 with L-glutamine, Penicillin-Streptomycin, FetalPlex™ Animal Serum Complex).

### Metal exposures

Sodium arsenite and cadmium chloride were purchased from Sigma (St. Louis, MO). Human TK6 cells were plated at 1 × 10^6 ^cells/ml and treated with 0.1 μM of either sodium arsenite or cadmium chloride for 24 hours. These equimolar, nonlethal doses were equivalent to 7.5 ppb of arsenic and 11.2 ppb of cadmium. Cell survival was assessed with the Trypan Blue Exclusion Assay using a hemocytometer with a 1:1 cell dilution and a 1:5 Trypan Blue dilution. For the arsenic exposures, two separate biological treatment replicates and two mock-treated controls were performed for subsequent microarray analysis. Likewise, for the cadmium exposures, two separate biological treatment replicates and two mock-treated controls were performed for subsequent microarray analysis.

### Microarray Processing

Total RNA was isolated from either arsenic or cadmium treated and mock treated human TK6 cells using the Qiagen's RNeasy^® ^Kit (Qiagen, Valencia CA). RNA was quantified with the NanoDrop™ 1000 Spectrophotometer (Thermo Scientific, Waltham MA) and its integrity was verified by the Agilent 2100 Bioanalyzer. RNA was biotin-labelled according to the Affymetrix protocol and hybridized to Affymetrix GeneChip^® ^Human Gene 1.0 ST arrays.

### Differential Gene Expression Analysis

Data were first normalized using Robust Multi-Chip Average (RMA) [58] and filtered for background noise (> abs[30]), which resulted in a reduction of probesets from 28869 to 20695 for arsenic and 21450 for cadmium. Differential gene expression was determined as a significant difference in the expression of a gene (exposed versus unexposed) where p < 0.01 (ANOVA model, Partek). Network analyses were performed using the Ingenuity software http://www.ingenuity.com. Statistical analyses were carried out within Ingenuity on both the arsenic and cadmium-modulated gene lists to identify the most significantly altered biological functions. Gene set enrichment analysis (GSEA) [17] was performed using the GSEA desktop software [59]. Significance is defined by the family wise error rate (FWER) p-value, which describes the probability that results are falsely positive. Microarray data have been submitted to the Gene Expression Omnibus (GEO) repository (GSE20320).

### Transcription Factor-binding Site Analysis

Transcription factor binding site analysis was performed using the EXPANDER 2.0 Software http://acgt.cs.tau.ac.il/expander/. For the analysis, Affymetrix probesets were linked to sequence data for regions 1,000 base pairs upstream and 200 base pairs downstream of the transcription start sites, and these were analyzed for significant enrichment of transcription factor binding sites. Significance (p ≤ 0.016) was calculated where significance is the probability of obtaining an equal or greater number of sequences with a model match in a randomly drawn sample of the same size as the input sequence set.

### Quantitative Real Time PCR Verification of Expression Data

Expression levels of the selected genes within the interactomes and sub-networks were validated with quantitative real time PCR (qRT-PCR) using RNA obtained from separate biological exposures (e.g. independent experiments) as those used for the microarray analyses. The forward and reverse primers were designed using Integrated DNA Technologies' Real Time PCR SciTool http://www.IDTdna.com/SciTools. The Qiagen QuantiTect SYBR Green PCR kit and Roche Lightcycler 480 were used for all qRT-PCR experiments. Fold changes between treated and untreated samples were calculated based on delta-delta cycle threshold (ddCt) values and normalized with β-actin as a housekeeping gene. Significance was calculated using a t-test.

## Authors' contributions

RCF conceived and designed experiments. LS and MAB performed exposures and determined cell killing. LS, JR, and MAB processed samples for microarray experiments. RCF, JR, and MAB analyzed the data. Manuscript was written by MAB, RCF, JR, and LS. All authors read and approved the final manuscript.

## Author Information

RCF is Assistant Professor at Gillings School of Global Public Health in the Department of Environmental Sciences and Engineering, University of North Carolina-Chapel Hill, MHRC 1213, CB#7431, Phone: 919-843-6864, Fax: 919-966-7911.

## Supplementary Material

Additional file 1 **Arsenic-modulated genes**. Additional File 1 lists all 74 probesets (62 genes) that showed significantly altered transcript levels resulting from arsenic exposure (0.1 μM for 24 hours). Corresponding gene symbols, reference sequences IDs, and fold changes are included. Click here for file

Additional file 2 **Cadmium-modulated genes**. Additional File 2 lists all 135 probesets (105 genes) that showed significantly altered transcript levels resulting from cadmium exposure (0.1 μM for 24 hours). Corresponding gene symbols, reference sequences, and fold changes are included. Click here for file

Additional file 3 **Gene products in networks**. Additional File 3 lists all of the proteins contained within the arsenic and cadmium-induced interactomes. Each protein is listed as either directly altered resulting from arsenic or cadmium exposure or associated with the directly altered transcripts. Also, proteins that are cancer-related are identified. Click here for file

Additional file 4 **Metal-modulated sub-networks**. Additional File 4 shows eight additional metal-modulated sub-networks. Arsenic sub-networks include (A) TNF-alpha associated sub-network 2 and (B) HNF-4 associated sub-network 3. Cadmium sub-networks include (C) p53 associated sub-network 1, (D) MYC-associated sub-network 3, (E) NF-kB associated sub-network 4, and (F) PI3K associated sub-network 5. Networks are displayed with symbols representing encoded proteins corresponding to their RNA transcripts that were either directly up-regulated (red symbols), down-regulated (green symbols), or associated with the modified transcripts (while symbols). Arsenic and cadmium gene sets were combined and mapped to (G) p38 MAPK associated sub-network 1, and (H) HNF-4 associated sub-network 3. Networks are displayed with symbols representing gene products of arsenic-modulated genes (red symbols) and cadmium-modulated genes (green symbols). Click here for file

Additional file 5 **Genes with enriched transcription factor binding sites**. Metal-modulated genes and their predicted transcription factors are listed. Click here for file

Additional file 6 **Comparison of gene expression levels of target genes assessed with qRT-PCR and microarray**. Fold changes in transcript levels for (A) arsenic and (B) cadmium-exposed samples are plotted for selected target genes. Correlations (R) between microarray and qRT-PCR values are displayed. Click here for file
