# Supplementary figures and images for: Comparative genomic analyses identify common molecular pathways modulated upon exposure to low doses of arsenic and cadmium
Source: BMC Genomics. 2011 Apr 1;12:173. doi: 10.1186/1471-2164-12-173 (PMC3082247; doi:10.1186/1471-2164-12-173)

## Additional File 4: Metal modulated sub-networks

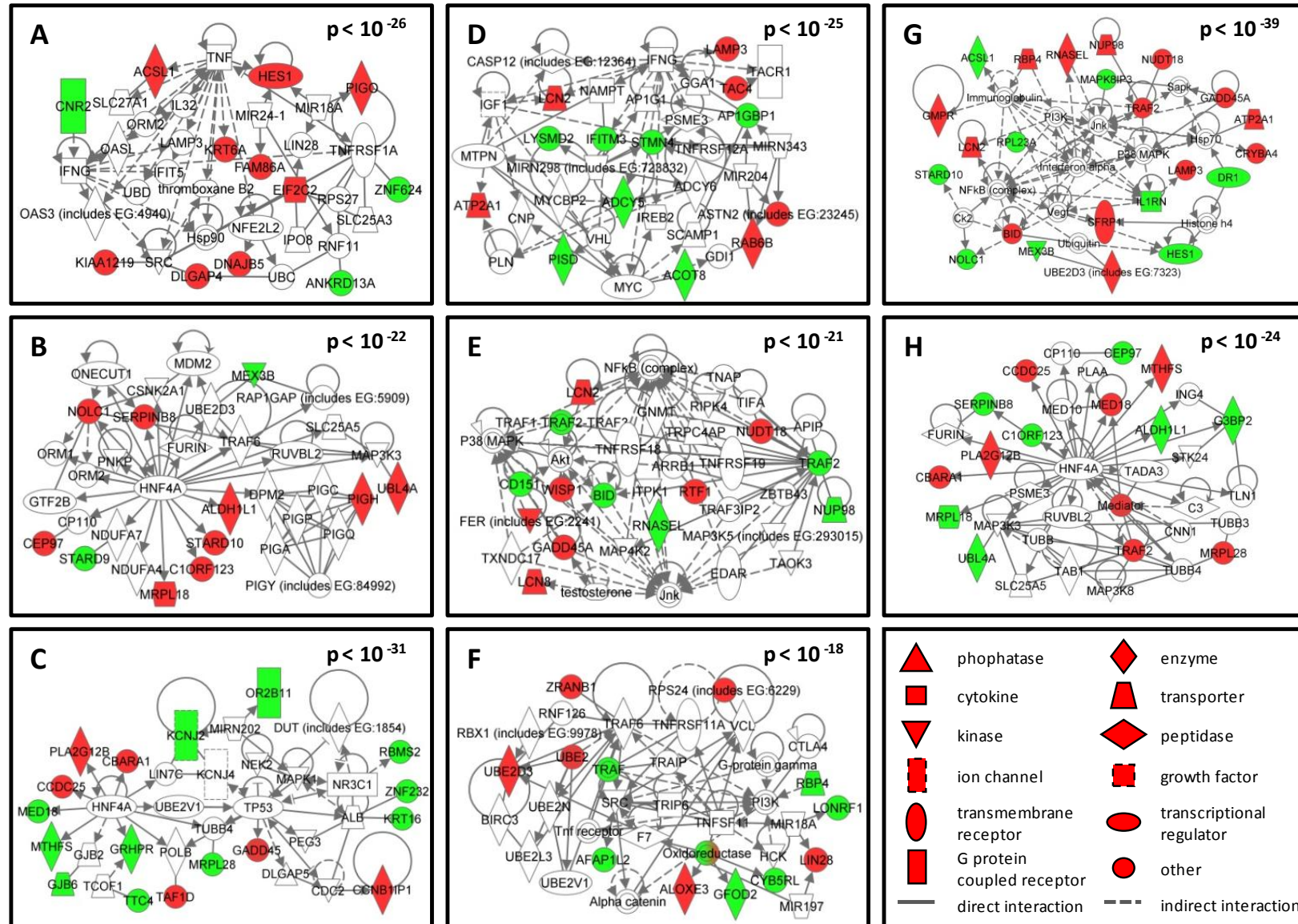

Supplement: Additional file 4 — Metal-modulated sub-networks. Additional File 4 shows eight additional metal-modulated sub-networks. Arsenic sub-networks include (A) TNF-alpha associated sub-network 2 and (B) HNF-4 associated sub-network 3. Cadmium sub-networks include (C) p53 associated sub-network 1, (D) MYC-associated sub-network 3, (E) NF-kB associated sub-network 4, and (F) PI3K associated sub-network 5. Networks are displayed with symbols representing encoded proteins corresponding to their RNA transcripts that were either directly up-regulated (red symbols), down-regulated (green symbols), or associated with the modified transcripts (while symbols). Arsenic and cadmium gene sets were combined and mapped to (G) p38 MAPK associated sub-network 1, and (H) HNF-4 associated sub-network 3. Networks are displayed with symbols representing gene products of arsenic-modulated genes (red symbols) and cadmium-modulated genes (green symbols). [file 1471-2164-12-173-S4.PDF]

Additional File 6: Comparison of qRT-PCR and microarray results

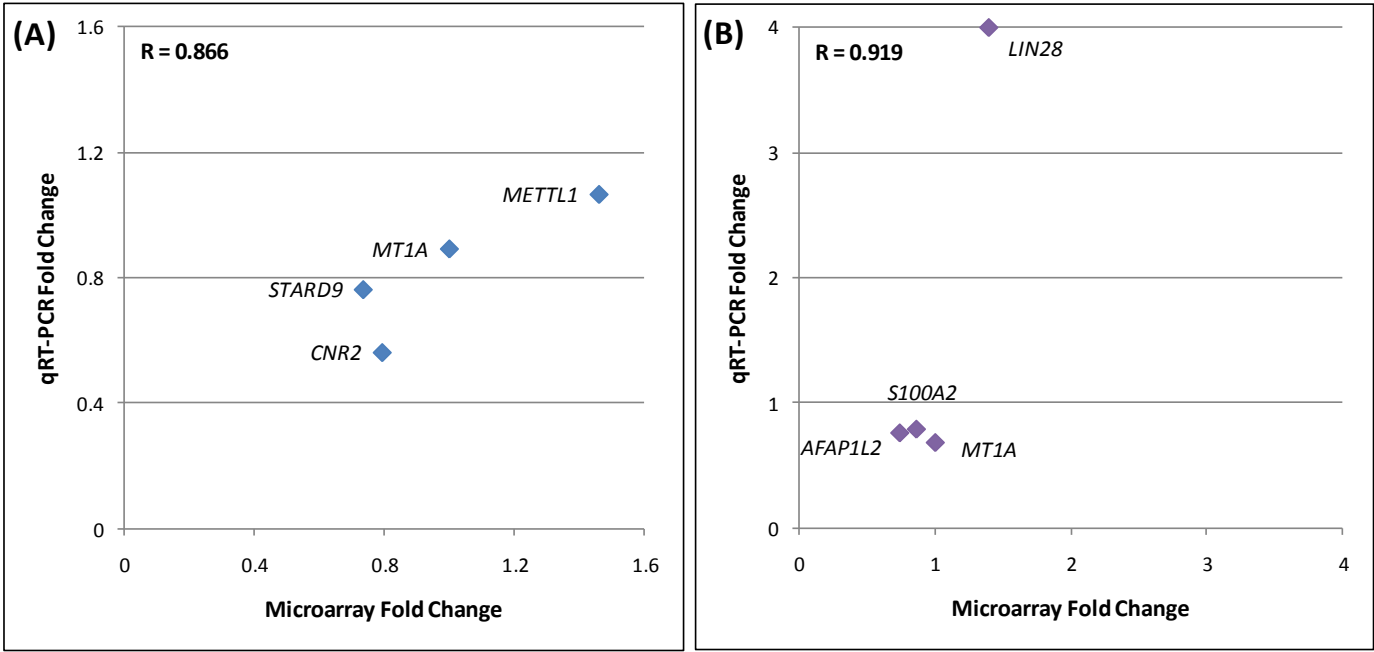

Supplement: Additional file 6 — Comparison of gene expression levels of target genes assessed with qRT-PCR and microarray. Fold changes in transcript levels for (A) arsenic and (B) cadmium-exposed samples are plotted for selected target genes. Correlations (R) between microarray and qRT-PCR values are displayed. [file 1471-2164-12-173-S6.PDF]
